# Sugammadex and rocuronium-induced anaphylaxis

**DOI:** 10.1007/s00540-015-2105-x

**Published:** 2015-12-08

**Authors:** Tomonori Takazawa, Hiromasa Mitsuhata, Paul Michel Mertes

**Affiliations:** Department of Anesthesiology, Gunma University Graduate School of Medicine, 3-39-22 Showa-machi, Maebashi, 371-8511 Japan; Department of Anesthesiology, Juntendo Tokyo Koto Geriatric Medical Center, 3-3-20 Shinsuna, Koto-Ku, Tokyo, 136-0075 Japan; Service d’anesthésie-réanimation chirurgicale, Nouvel hôpital civil, hôpitaux universitaires de Strasbourg, 1, place de l’Hôpital, BP 426, 67091 Strasbourg Cedex, France

**Keywords:** Sugammadex, Rocuronium, Anaphylaxis, Neuromuscular blocking agent, Flow cytometry

## Abstract

Perioperative anaphylaxis is a life-threatening clinical condition that is typically the result of drugs or substances used for anesthesia or surgery. The most common cause of anaphylaxis during anesthesia is reportedly neuromuscular blocking agents. Of the many muscle relaxants that are clinically available, rocuronium is becoming popular in many countries. Recent studies have demonstrated that succinylcholine (but also rocuronium use) is associated with a relatively high rate of IgE-mediated anaphylaxis compared with other muscle relaxant agents. Sugammadex is widely used for reversal of the effects of steroidal neuromuscular blocking agents, such as rocuronium and vecuronium. Confirmed cases of allergic reactions to clinical doses of sugammadex have also been recently reported. Given these circumstances, the number of cases of hypersensitivity to either sugammadex or rocuronium is likely to increase. Thus, anesthesiologists should be familiar with the epidemiology, mechanisms, and clinical presentations of anaphylaxis induced by these drugs. In this review, we focus on the diagnosis and treatment of anaphylaxis to sugammadex and neuromuscular blocking agents. Moreover, we discuss recent studies in this field, including the diagnostic utility of flow cytometry and improvement of rocuronium-induced anaphylaxis with the use of sugammadex.

## Introduction

Sugammadex is a synthetic γ-dextrin derivative that was first designed to selectively bind to the steroidal neuromuscular blocking agent (NMBA) molecule. Sugammadex is now available in over 60 countries, including the European Union, Australia, and Japan. However, it has not yet been approved by the Food and Drug Administration in the USA due to concerns regarding hypersensitivity. Sugammadex has approximately 2.5 times the affinity and selectivity for rocuronium than for vecuronium [1]. However, it has no affinity for succinylcholine or benzylisoquinoline nonsteroidal muscle relaxants. Thus, reversal by sugammadex is an incentive to favor the use of steroidal instead of nonsteroidal muscle relaxants. In addition, possible rapid reversal is an incentive to propose the use of rocuronium instead of succinylcholine for rapid sequence induction. For these reasons, use of the combination of rocuronium and sugammadex is becoming popular in some countries, including Japan. However, since rocuronium is also reportedly associated with a higher rate of IgE-mediated anaphylaxis compared with other steroidal NMBAs [2, 3], the probability of the number of cases of hypersensitivity to either drug increasing is high. In this review, we seek to highlight the current knowledge regarding the epidemiology, mechanisms, clinical presentation, diagnosis, and treatment of sugammadex and rocuronium-induced anaphylaxis.

## Epidemiology

### Intraoperative anaphylaxis

Anaphylaxis is defined as “a serious allergic reaction that has a rapid onset and may cause death” [4]. The rate of anaphylaxis has increased during the last decade. This increase is reportedly as high as 350 % for food-induced anaphylaxis and 230 % for nonfood-induced anaphylaxis over the last decade [5]. Regarding an immediate allergic hypersensitivity reaction during anesthesia, its incidence rate varies between different countries from 1/10,000 to 1/20,000 [6]. Between 2009 and 2011, the Japanese Society of Anesthesiologists (JSA) conducted a survey on intraoperative complications and reported a total of 237 cases of anaphylaxis during anesthesia. The incidence of anaphylaxis based on this survey was approximately 1/18,600. This incidence included 13 cases of cardiac arrest and one fatal case. Anaphylaxis was the most common cause of complications during anesthesia that was independent of surgery, anesthetic management, and pre-existing comorbidities (data are available for JSA members at the following URL; https://member.anesth.or.jp/App/datura/news2013/pdf/r20130503.pdf). Hence, although all anesthesiologists may not experience cases of intraoperative anaphylaxis, it is important for them to be aware of the possibility of intraoperative anaphylaxis and prepare appropriate drugs and devices for its treatment available.

### NMBA-induced anaphylaxis

In France, between 2005 and 2007, the most common cause of anaphylaxis during anesthesia was reportedly NMBAs (47.4 %). This was followed by latex (20 %) and antibiotics (18.1 %) [7]. Succinylcholine (60.6 %) and atracurium (19.6 %) were the major causative drugs, whereas anaphylaxis to cisatracurium (5.9 %), vecuronium (4.6 %), rocuronium (4.3 %), pancuronium (2.7 %), and mivacurium (1.9 %) was also reported [7]. However, these results were at the origin of controversy because of the difficulty in obtaining definite information concerning the number of patients exposed to each compounds. Denominator based on cases actually exposed to each agent is hard to obtain because of the difficulties associated with retrieval of the administration records of many thousands of anesthetics [3]. For this reason, relevant denominators have usually been estimated from sales data or similar metrics, which, however, fail to account for confounders such as vials opened but not used, expired vials, and repeat administrations or infusions. The variation in the reported incidence of anaphylaxis to rocuronium, approximately 1:3500–1:445,000 [8, 9], could be explained by these biases. To overcome this problem, a study with accurate numerators and denominators for the calculation was recently conducted at two hospitals in Auckland, New Zealand [3]. This study demonstrated that the rate of anaphylaxis due to succinylcholine, rocuronium, and atracurium was 1:2079, 1:2498, and 1:7680–109,000, respectively. Although the risk of allergic reactions is not the only drug characteristic that anesthesiologists must take into account when making their clinical choice, the likely increased allergic risk associated with succinylcholine and rocuronium, and the relatively low risk with atracurium must be part of the clinical reasoning when considering the use of a NMBA [10]. Cisatracurium had the lowest rate of cross-reactivity in patients who had previously suffered anaphylaxis to rocuronium and vecuronium [2]. Therefore, anesthesiologists should consider the use of cisatracurium as an alternative safe agent, if it is available.

### Sugammadex-induced anaphylaxis

Confirmed cases of allergic reactions to clinical doses of sugammadex have been recently reported [11, 12]. However, the number of reports of sugammadex-induced anaphylaxis is much less than those for NMBAs [12, 13]. To date, the Japanese Society of Anesthesiologists has issued a warning about sugammadex-induced anaphylactic shock five times since March 2011. The third one, issued in June 2013, included 95 cases of sugammadex-related allergies that occurred between April 2010 and January 2013, although with no incidents of death. In all 95 cases, the relationship between the reaction and sugammadex was definitively ascertained by the attending anesthesiologists. Seventy-eight of the 95 cases fulfilled the validation criteria for anaphylaxis. The incidence rate of anaphylactic reactions due to sugammadex was estimated as 29 per million cases (1:34,483), based on the estimated number of patients (3.09 millions) in whom sugammadex was injected during the survey period. The alert also pointed out that the incidence rate may have been underestimated, because the survey was based on spontaneous reports from anesthesiologists and not on prospective studies. It is uncertain whether this incidence rate is higher in Japan than in other countries, because there is no epidemiological survey regarding this so far. The other possibility is that these warnings may simply reflect a high level of sugammadex usage in Japan. The drug company, MSD (Tokyo, Japan), has reported that sugammadex usage in Japan in 2010, in terms of monetary value, was more than four times higher than that in Spain, the country that showed the second-highest usage in the world [11].

## Mechanisms

Anaphylaxis mediated by IgE, IgG, complement, or immune complex is defined as immune-mediated anaphylaxis, as opposed to non-allergic anaphylaxis (previously known as an anaphylactoid reaction) [14]. Allergic reactions to NMBAs are almost exclusively IgE-mediated. Up to 75 % of reactions have been reported upon first known contact with the NMBA [15, 16]. This suggests a possible cross-reaction with IgE antibodies generated by previous contact with apparently unrelated chemicals.

### Pholcodine hypothesis

This hypothesis originated from the literature that pointed out that anaphylaxis to NMBAs was ten times more common in Norway than in Sweden [17]. The authors suggested that this difference could be due to differences in preoperative sensitization due to pholcodine consumption. Indeed, cough syrups containing pholcodine were available in Sweden during the 1970s and 1980s, but were thereafter withdrawn from the market [18]. Further studies conducted to support this hypothesis showed that withdrawal of pholcodine from the Norwegian market significantly lowered IgE antibodies to pholcodine, morphine, and suxamethonium, and even the frequency of suspected NMBA anaphylaxis [19]. However, it is unknown whether the decrease in the frequency of suspected NMBA anaphylaxis was actually a result of lowering of these IgE antibodies. One could argue that an unknown environmental factor, other than pholcodine, may have caused this “co-incidence”. In fact, IgE sensitization to pholcodine and morphine was prevalent even in low pholcodine-consuming countries, such as the USA and the Netherlands [20], suggesting that IgE sensitization to pholcodine may even occur in the absence of pholcodine. In other words, other compounds may lead to production of IgE which will cross react with pholcodine, NMBAs, and other quaternary ammonium-containing compounds. Further epidemiological studies to investigate the possible link between pholcodine exposure and hypersensitivity reactions to NMBAs are required.

In contrast to NMBA-mediated anaphylaxis, little is known about the mechanism of sugammadex-mediated anaphylaxis. It is unknown whether the hypersensitivity reactions to sugammadex are IgE-mediated or non-IgE-mediated due to the lack of sugammadex-specific IgE antibodies. Direct exposure of the patient’s blood to sugammadex may induce non-specific release of various chemical mediators, such as histamine and tryptase, from peripheral tissue-resident mast cells, although there is no evidence that such a phenomenon does actually occur. Indeed, a recent study identified a mast cell-specific receptor that is responsible for non-allergic anaphylaxis [21]. In 15 cases reported in the literatures, none of the patients had a history of previous exposure to sugammadex [12]. This evidence suggests cross-reactivity between sugammadex and some unknown substance. One such possible candidate substance is cyclodextrin, present in foods and/or drugs. In fact, the average person consumes approximately 4 g of gamma-cyclodextrins per day in foods [22].

## Clinical presentation

In general, anaphylaxis symptoms involve several organ systems, including the skin, causing mainly urticarial (80–90 % of episodes), respiratory tract (70 % of episodes), gastrointestinal tract (30–45 % of episodes), cardiovascular (10–45 % of episodes), and central nervous system (10–15 % of episodes) [23–26]. Cardiovascular symptoms, including hypotension and bradycardia, are more common during events occurring in the operating room and are associated mainly with the use of muscle NMBAs and latex [24]. It is easy for anesthesiologists to recognize changes in the hemodynamic conditions of patients (i.e., blood pressure and heart rate) because they are usually monitored in the operation room. When a patient develops respiratory symptoms, such as bronchospasm, tracheal intubation is often required. In some cases of NMBA-induced anaphylaxis, symptoms may appear after tracheal intubation. However, in case of rapid appearance of symptoms, the anesthesiologist may become aware of difficulty in mask ventilation. Sugammadex-induced anaphylaxis typically presents when the patient is already extubated and is being transferred to their hospital bed, the PACU, or ICU, etc. [27]. In these cases, emergency re-intubation outside the operating room may be required. Skin involvement with anaphylaxis may be less frequent in perioperative reactions, making the diagnosis more difficult [28]. In addition to being less frequent, skin manifestations are also not easily recognized because the patient is covered and sedated, being unable to report pruritus. Moreover, hoarseness, dysphagia, dizziness, and blurred vision, which are warning signs of severe anaphylactic reactions, will not be present in a sedated patient [28].

## Diagnosis

### Clinical signs

The first line of evidence for diagnosing anaphylaxis includes clinical signs. The National Institute of Allergy and Infectious Diseases (NIAID) and the Food Allergy and Anaphylaxis Network (FAAN) proposed clinical criteria for diagnosing anaphylaxis [4]. Anaphylaxis is considered likely if any one of three stipulated criteria is satisfied within minutes to hours (Table 1). Depending on the severity of the reaction, four grades of immediate clinical manifestations are described: Grade 1, Cutaneous signs only; Grade 2, Measurable but not life-threatening symptoms and cutaneous signs, hypotension, tachycardia, and respiratory disturbances, such as cough and difficulty in lung inflation; Grade 3, Life-threatening symptoms: collapse, tachycardia or bradycardia, arrhythmias, bronchospasm; Grade 4, Cardiac and/or respiratory arrest [29].

**Table 1:** Clinical criteria for diagnosing anaphylaxis

Anaphylaxis is highly likely when any one of the following three criteria are fulfilled:
1. Acute onset of an illness (over minutes to several hours) with involvement of the skin, mucosa, or both (e.g., generalized hives, pruritus or flushing, swollen lips-tongue-uvula)
And at least one of the following
(a) Respiratory compromise (e.g., dyspnea, wheeze-bronchospasm, stridor, reduced PEF, hypoxemia)
(b) Reduced BP or associated symptoms of end-organ dysfunction [e.g., hypotonia (collapse), syncope, incontinence]
2. Two or more of the following that occur rapidly after exposure to a likely allergen for that patient (within minutes to several hours):
(a) Involvement of the skin-mucosal tissue (generalized hives, itch-flush, swollen lips-tongue-uvula)
(b) Respiratory compromise (e.g., dyspnea, wheeze-bronchospasm, stridor, reduced PEF, hypoxemia)
(c) Reduced BP or associated symptoms (e.g., hypotonia [collapse], syncope, incontinence)
(d) Persistent gastrointestinal symptoms (e.g., cramping abdominal pain, vomiting)
3. Reduced BP after exposure to a known allergen for that patient (within minutes to several hours):
(a) Infants and children: low systolic BP (age specific) or greater than 30 % decrease in systolic BP^a^
(b) Adults: systolic BP of less than 90 mmHg or greater than 30 % decrease from that person’s baseline

Modified from Sampson et al. [4]

PEF, peak expiratory flow; BP, blood pressure

^*^Low systolic blood pressure for children is defined as less than 70 mmHg from 1 month to 1 year, less than [70 mmHg + (2× age)] from 1 to 10 years, and less than 90 mmHg from 11 to 17 years

It is known that the onset of perioperative anaphylaxis usually occurs within 5 min after induction of anesthesia [30, 31]. Thus, the timing of appearance of clinical signs sometimes aids in diagnosis. However, agents that are administered via other routes, e.g., those applied on the skin and mucosa, in the urethra, contact with the peritoneum or subcutaneously, may take some time to be absorbed and may, therefore, cause reactions after more than 15 min [31]. In addition, physicians often need to discriminate between anaphylaxis and other diseases, including pulmonary embolism, asthma, and seizure disorders.

### Laboratory tests

The second line of evidence for diagnosing anaphylaxis is biological assessment, including plasma histamine and tryptase measurements [32, 33]. Plasma histamine levels are increased for only 15–60 min after symptom onset. In addition, special handling of the blood sample is required, for example, obtaining it through a wide-bore needle, keeping it cold at all times, centrifuging it immediately, and freezing the plasma promptly [33, 34]. For isolated mucocutaneous (grade 1) reactions, the delay in blood sampling should ideally be less than 15 min after the reaction, for grade 2 reactions sampling should be performed within 30 min, and within 2 h for more severe reactions [6]. Plasma or serum total tryptase levels, on the other hand, are increased from 15 min to 3 h after symptom onset, and its measurement requires no special handling of the blood sample. Typically, anaphylaxis results from mast cell activation, which causes release of mast cell tryptase into circulation, although a variety of other pathways, including basophil or complement activation, may combine to produce anaphylaxis. These tests have limitations when used to confirm the diagnosis of an acute anaphylaxis episode, including suboptimal specificity and sensitivity. Thus, the diagnostic accuracy of these assays is increased when measurement of histamine and tryptase are combined [6, 32]. Measurement of allergen-specific IgE levels in serum is also helpful in patients who have experienced anaphylaxis. Specific IgE antibodies against succinylcholine (thiocholine ester) can be assayed in serum, although the sensitivity is relatively poor (30–60 %). Moreover, test kits for serum IgE antibodies against other NMBAs but sugammadex are also commercially available [35].

### Skin tests

Skin tests, the third line of evidence, remain the gold standard for detection of IgE-mediated reactions, and involve exposure of the mast cells in the skin of patients who experience anaphylaxis to the suspected allergen [36]. The main risk factor for perioperative anaphylaxis to anesthetic drugs is a previously uninvestigated severe immediate hypersensitivity reaction during the perioperative period [6]. Therefore, an allergological assessment should be performed prior to the surgical procedure, if possible. Anesthesiologists usually use multiple drugs, including hypnotics, NMBAs and opioids, for the induction of anesthesia. Thus, it is essential to perform skin tests to several agents when NMBAs are suspected as being the causative agents of anaphylaxis. Skin prick and/or intradermal tests are usually performed 4–6 weeks after the acute reaction [6]. A histamine solution (10 mg/ml for skin prick test and 10 μg/ml for intradermal test) and physiological saline are used as positive and negative controls, respectively. The sensitivity of the skin prick test seems to be inferior to that of the intradermal test for most drugs [6]. Yet, it is preferable to perform skin prick test prior to intradermal test for NMBAs and sugammadex, since the results of the skin prick test help in determining the first concentration of the drug tested with the intradermal test. When the skin prick test is negative, intradermal test starts at a 1/1000 dilution of the stock solution for NMBAs. The optimal concentration of both skin prick and intradermal test for specific drugs, including NMBAs, has been previously shown [37, 38]. For example, the maximum concentration of rocuronium for skin prick and intradermal test are 10 mg/ml and 100 μg/ml, respectively. Prior to proceeding with skin test studies on any drug, testing for specificity and optimal test concentrations should be undertaken [39]. However, validation studies of the skin prick and intradermal test for sugammadex currently appear to be lacking, except for one recent study, which showed that 1:77 and 1:770 dilutions of 100 mg/ml sugammadex for intradermal tests did not cause skin irritation or false-positive reactions in 11 volunteers, suggesting that 1:100 and higher dilutions of sugammadex are not likely to produce false-positive reactions [40]. Further studies are still needed to clarify the optimal drug concentration during skin prick and intradermal test for sugammadex.

### Flow cytometry

The flow cytometry-assisted basophil activation test (BAT) has been utilized in the diagnosis of immediate-type drug hypersensitivity since the early 1990s, when CD63 was discovered as a marker of basophil activation [41]. This assay has the advantage of not being associated with the risk of inducing anaphylaxis during the test [42]. Another advantage of the BAT is the relatively high sensitivity and specificity for identification of NMBA-induced anaphylaxis. In fact, the sensitivity and specificity of BAT for rocuronium-induced anaphylaxis were reportedly 91.7 and 100 %, respectively [43]. The diagnostic utility of BATs in immediate type NMBA hypersensitivity was summarized in a previous review. According to this review, its specificity was nearly 100 % in most studies, whereas sensitivity was in the range of 40–90 % [44]. Given the high specificity and mediocre sensitivity, the combination of multiple methods, such as skin tests and BAT, is likely to improve the diagnostic precision for anaphylaxis. So far, there has been only one report describing the usefulness of BAT for diagnosing sugammadex-induced anaphylaxis [42].

## Treatment

**Fig. 1:**
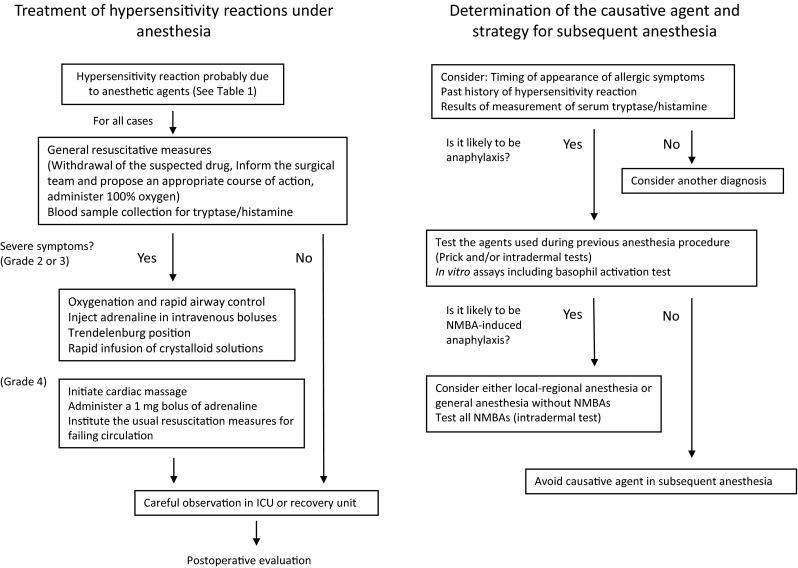
Anesthetic management of patients with perioperative anaphylaxis (diagnosis, treatment, and strategy for subsequent anesthesia). Modified from Mertes et al. [6]

Quick recognition of anaphylactic signs and symptoms is essential for a favorable prognosis. In this respect, anesthesiologists have certain advantages since: (1) hemodynamics are usually monitored in patients under general anesthesia, and (2) an intravenous line for administration of drugs to treat anaphylaxis has already been established. There are several guidelines showing how physicians should treat a patient who has developed symptoms that are likely to be caused by anesthetic drugs during anesthesia [4, 6, 31, 45] (Fig. 1). Adrenaline is the only drug recommended as first-line therapy in all published national anaphylaxis guidelines. However, the guidelines do not agree on the initial dose or route of injection of epinephrine. It should be emphasized that treatment must be tailored according to the clinical severity, patient’s history, and availability of and response to emergency treatment [6].

Primary treatment other than drug therapy is as follows: (1) stop administration of the suspected substance; (2) call for help and inform the surgeon; (3) apply the Trendelenburg position; and (4) maintain the airway and give oxygen [31]. Secondary treatment includes administration of corticosteroids and antihistamines. Although corticosteroids may attenuate the late manifestations of shock [6], there is no evidence from high-quality studies for the use of steroids in the emergency management of anaphylaxis [46]. There is also no consensus among published national anaphylaxis guidelines with regard to the use of antihistamines [34]. In cases with bronchospasm without arterial hypotension, an inhaled β_2_-adrenergic receptor agonist, such as salbutamol or nebulized adrenaline, should be administered [6, 31, 47]. On rare occasions, adrenaline appears to be ineffective in anaphylaxis. In such cases, drugs such as noradrenaline, vasopressin, and glucagon are recommended [6, 31, 45].

### Can sugammadex improve rocuronium-induced anaphylaxis?

Several past reports have suggested the possibility of improvement in rocuronium-induced anaphylaxis by the administration of sugammadex [48–51], based on the speculation that by encapsulating rocuronium, sugammadex might offer a novel treatment to reverse anaphylaxis caused by NMBAs. Central to this hypothesis is whether or not the allergenic ammonium groups of NMBAs are still able to interact with the complementary IgE-antibodies once the NMBA is encapsulated [52, 53]. Another issue is whether or not encapsulation of rocuronium by sugammadex may prevent further mediator release from the mast cells and basophils. Some investigators performed laboratory experiments to answer these questions [53–56]. Molecular models indicated that the determinants of hypersensitivity may still be accessible to binding [53, 54]. In addition, CD63 expression, a marker of basophil activation, could not be blocked when sugammadex was added after basophils had already been activated by rocuronium [55]. Studies using a cutaneous model similarly concluded that sugammadex is unlikely to significantly modify the clinical course of an established allergic reaction [40]. In contrast to these evidences from laboratory settings, a review that summarized 11 cases from seven different countries demonstrated recovery from anaphylaxis after sugammadex administration [13]. Some authors believe that the timing of recovery in relation to the drug’s administration and the rapidity and extent of recovery support the efficacy of sugammadex [50]. There are several hypotheses to explain the discrepancy between evidences from clinical and laboratory settings, including that administration of sugammadex and alleviation of symptoms may have coincided with the beneficial effects of the already-instituted therapy with adrenaline injection and fluid resuscitation [48]. A recent report that retrospectively analyzed 13 cases of presumed rocuronium-induced anaphylaxis concluded that sugammadex does not modify the clinical course of a suspected hypersensitivity reaction [57]. The authors raised the hypothesis that sugammadex administration on anaphylaxis improves cardiac preload after increasing muscle tone by reversing neuromuscular blockade. Moreover, they cautioned against including sugammadex in anaphylaxis-treatment algorithms [57]. Further studies may be still needed to put an end to this debate.

### Anesthetic management of a patient with a history of anaphylaxis during previous surgery

As patients with NMBA anaphylaxis frequently cross-react with other NMBAs, alternative anesthetic techniques that do not require the use of muscle relaxants should be considered for subsequent operations. If this is difficult, alternative safe NMBAs should be used. Rocuronium has an intermediate risk of anaphylaxis, as its rate of cross-reactivity is less than that of succinylcholine but greater than that of vecuronium [58] (Fig. 1). A study on the cross-reactivity between NMBAs demonstrated that patients with rocuronium anaphylaxis are most likely to also test positive for succinylcholine (44 %) and vecuronium (40 %) during skin tests, while pancuronium and atracurium were also frequent cross-reactors (19 and 20 %, respectively) [2]. Cisatracurium was the least likely to cross-react, at 5 % [2]. Given the possibility of false-negative reactions in skin tests, avoidance of the use of all NMBAs in patients with a history of anaphylaxis to rocuronium seems prudent. Indeed, there are several case reports describing patients in whom false-negative skin tests led to a second severe anaphylactic reaction to another NMBA [59, 60]. If the surgical procedure requires muscle relaxation, the anesthesiologist should assess the balance of risks. In the case of sugammadex-induced anaphylaxis, it is much easier to find an alternative safe drug, such as neostigmine, which is the classical reversal agent for NMBAs. In many aspects, sugammadex is superior to classical reversal agents. However, the demerits of neostigmine are negligible compared to the risk of anaphylaxis with sugammadex. Besides avoiding the anaphylaxis-inducing agent, prudent anesthetic management, including preparation of therapeutic agents and careful monitoring, is required for patients with a past history of perioperative anaphylaxis.
